# PI3 kinase mutations and mutational load as poor prognostic markers in diffuse glioma patients

**DOI:** 10.1186/s40478-015-0265-4

**Published:** 2015-12-23

**Authors:** Kaspar Draaisma, Maarten M. J. Wijnenga, Bas Weenink, Ya Gao, Marcel Smid, P. Robe, Martin J. van den Bent, Pim J. French

**Affiliations:** Department of Neurology, Erasmus MC, Room Be 430A, POBox 2040, 3000 CA Rotterdam, The Netherlands; Department of Medical Oncology, Erasmus MC, Rotterdam, The Netherlands; Department of Neurosurgery, UMC Utrecht, Utrecht, Netherlands; Department of Human Genetics, University of Liège, Liège, Belgium

**Keywords:** Diffuse glioma, PIK3CA, PIK3R1, Mutational load, Tumor grade, IDH1, IDH2, 1p19q codeletion, ATRX, TP53

## Abstract

**Introduction:**

Recent advances in molecular diagnostics allow diffuse gliomas to be classified based on their genetic changes into distinct prognostic subtypes. However, a systematic analysis of all molecular markers has thus far not been performed; most classification schemes use a predefined and select set of genes/molecular markers. Here, we have analysed the TCGA dataset (combined glioblastoma (GBM) and lower grade glioma (LGG) datasets) to identify all prognostic genetic markers in diffuse gliomas in order to generate a comprehensive classification scheme.

**Results:**

Of the molecular markers investigated (all genes mutated at a population frequency >1.7 % and frequent chromosomal imbalances) in the entire glioma dataset, 57 were significantly associated with overall survival. Of these, *IDH1* or *IDH2* mutations are associated with lowest hazard ratio, which confirms *IDH* as the most important prognostic marker in diffuse gliomas. Subsequent subgroup analysis largely confirms many of the currently used molecular classification schemes for diffuse gliomas (*ATRX* or *TP53* mutations, 1p19q codeletion). Our analysis also identified *PI3*-kinase mutations as markers of poor prognosis in *IDH*-mutated + *ATRX/TP53* mutated diffuse gliomas, median survival 3.7 *v*. 6.3 years (*P* = 0.02, Hazard rate (HR) 2.93, 95 % confidence interval (CI) 1.16 – 7.38). *PI3-*kinase mutations were also prognostic in two independent datasets. In our analysis, no additional molecular markers were identified that further refine the molecular classification of diffuse gliomas. Interestingly, these molecular classifiers do not fully explain the variability in survival observed for diffuse glioma patients. We demonstrate that tumor grade remains an important prognostic factor for overall survival in diffuse gliomas, even within molecular glioma subtypes. Tumor grade was correlated with the mutational load (the number of non-silent mutations) of the tumor: grade II diffuse gliomas harbour fewer genetic changes than grade III or IV, even within defined molecular subtypes (e.g. *ATRX* mutated diffuse gliomas).

**Conclusion:**

We have identified PI3K mutations as novel prognostic markers in gliomas. We also demonstrate that the mutational load is associated with tumor grade. The increase in mutational load may partially explain the increased aggressiveness of higher grade diffuse gliomas when a subset of the affected genes actively contributes to gliomagenesis and/or progression.

**Electronic supplementary material:**

The online version of this article (doi:10.1186/s40478-015-0265-4) contains supplementary material, which is available to authorized users.

## Introduction

Gliomas are the most common primary malignant brain tumors in adults [1, 2]. Diffuse gliomas are classified into different subtypes according to their histological features into astrocytomas, oligodendrogliomas and mixed oligoastrocytomas [3]. These subtypes are further divided into various tumor grades (grade II-IV) depending on the number of malignant features present in the tumor (nuclear atypia, mitoses, endothelial proliferation and necrosis). The WHO classification, in combination with clinical parameters such as age and Karnofsky Performance Status (KPS), guides treatment decisions and provides prognostic information for patients and clinicians.

Unravelling the causal genetic changes of diffuse gliomas has been the focus of extensive research in the past decade [4–6] and it is now possible to classify diffuse gliomas based on their molecular characteristics [7–11]. For example, *IDH1* mutations are frequent events in all grade II and III gliomas and in secondary glioblastomas (sGBM, glioblastomas that progress from lower grade gliomas) whereas primary GBMs (pGBM) are usually *IDHwt* and frequently have genetic changes involving the *EGFR* locus, *PTEN* deletions and *TERT* promoter mutations [4, 6, 12]. In addition, *CIC, FUBP1, TERT* promoter mutations and 1p/19q codeletion are observed more frequently in oligodendrogliomas than in astrocytic tumors [13–15] whereas *ATRX* and *TP53* mutations are seen more frequently in grade II/III astrocytic tumors [16–18]. The importance of this molecular information is widely acknowledged and guidelines have been made to incorporate them in the WHO classification of gliomas [19].

Although the genetic changes are used to classify diffuse gliomas into distinct prognostic subtypes [9, 10, 16, 20–23], a systematic analysis of all available molecular prognostic markers has thusfar not been performed. In fact, most classification schemes use only a few high frequent genes or molecular markers. It is therefore possible that additional and/or stronger prognostic markers are present that can improve the molecular classification of diffuse gliomas. Furthermore, while the prognostic molecular markers may refine (or even replace) the histological classification of diffuse gliomas, there are thusfar no genetic changes that can discriminate between grade II and III tumors. This is remarkable as tumor grade is a strong prognostic marker in diffuse gliomas [3] (although some reports found little prognostic value for tumor grade within defined glioma subtypes [24, 25]).

In this study we therefore have analyzed the publicly available TCGA dataset in order to identify additional prognostic molecular markers in diffuse gliomas. Since diffuse gliomas can be classified solely based on molecular markers [9, 20], we also evaluated whether tumor grade remains relevant after the molecular classification and/or whether there are genetic markers that can distinguish between tumor grades in diffuse gliomas. Our analysis confirms many of the currently used molecular classification schemes for diffuse gliomas: gliomas are first separated based on *IDH-*mutation status and a further stratification is based on *ATRX/TP53* mutation status or 1p19q codeletion. We show that *PI3*-kinase mutations are associated with poor prognosis in molecular astrocytomas (i.e. diffuse gliomas that are *IDH*-mutated and 1p19q intact (or *ATRX*/*TP53* mutated)) and that no other marker investigated in this study appears to further refine this molecular/prognostic classification of diffuse gliomas. Our analysis also shows that, for most driver mutations investigated here (*IDH1/2, ATRX, TP53*), tumor grade remains a prognostic factor in diffuse gliomas with identical driver mutations. This indicates that *IDH*-mutated glioblastomas behave significantly more aggressive than *IDH*-mutated grade III gliomas. Although no single molecular marker was associated with tumor grade, we find that tumor grade is correlated with the overall mutational load: grade II gliomas harbour fewer genetic changes than grade III or IV, even within defined molecular subtypes (e.g. *ATRX* mutated gliomas). The increased mutational load may partially explain the increased aggressiveness of higher grade gliomas when a subset of the affected genes actively contribute to gliomagenesis and/or progression.

## Materials and methods

For this study, we have used publicly available data from the TCGA, both lower grade glioma and glioblastoma datasets. Data include mutation status, copy number variations and clinical data, only cases with complete data were included in current analysis (*n* = 542). All data analysis were based on overall survival (OS). Survival data for patients that are listed as <30 days were omitted from the survival analysis; the cause of death for such patients may not be tumor-related (but e.g. related to complications occurring after surgery). *EGFR* amplification status and *CDKN2A* deletions data were downloaded from the cbioportal site [26]. Although such data could be extracted from the copynumber data (see below), we used cBioportal data to ensure identical thresholds were used to define amplification and allelic loss. All mutation data were filtered for those that result in a change in the primary amino acid sequence. We focussed on all genes that are mutated in more than ten samples of the entire study population We also included the copy number alterations 1p19q codeletion (loss of heterozygosity (LOH) of the 1p and 19q chromosome arms) and trisomy of chromosome 7 and LOH of chromosome 10 (alt 7/10). Combined, we analysed 128 genetic alterations in 542 samples.

Genome wide SNP 6 Copynumber data was downloaded from the TCGA dataportal. This data gives a value per chromosomal region (segment) where values deviating from 0 likely correspond to regions with chromosomal losses (<0) or gains (>0). From the segment values, we calculated the average an entire chromosome/chromosomal arm and defined 1p19q codeletion as averages over both arms -0.3 or less. When values were disconcordant between 1p and 19q or values were between 0 and −0.3 (which can occur in tumors with a high content of non-neoplastic tissue), we determined 1p19q codeletion based on visualization of the copynumber plot. This visualization was performed blinded to the patient outcome. Alt 7/10 was determined by a value of 0.3 or higher for chromosome 7 *and* a value of −0.3 or lower for chromosome 10. When values were either discordant between chromosome 7 and 10, or were between 0 and 0.3 for chromosome 7 and/or between 0 and −0.3 for chromosome 10, we determined alt 7/10 based on visualization of the copynumber plot (blinded to patient outcome). Because *IDH1* and *IDH2* mutations are mutually exclusive and play an identical role in tumor pathogenesis, we have combined mutation data into an additional single *IDH*-mutations variable. Similarly, we combined *EGFR*-mutations and *EGFR* gene amplifications into a single additional *EGFR*-alteration variable. As *PIK3CA* and *PIK3R1* are highly related (and mutually exclusive) genes within the same PI3-kinase pathway, we also combined mutation data into an additional single *PI3-*kinase mutations variable.

To validate the prognostic value of identified genes, we performed survival analysis on two additional datasets containing mutation and survival data [6, 17]. Hazard ratios (HR) and survival differences were calculated using a cox proportional hazard model in R (survival CRAN package), unless specifically indicated otherwise. Differences in mutation frequencies were calculated using an ANOVA (3 groups) or *T*-Test (2 groups). Bonferroni correction was done by using a P value cutoff of 0.0004 (0.05 divided by the total number of calculations (128 genes and copy number changes)). Chi square tests were performed using an online calculator (www.quantpsy.org/chisq/chisq.htm), Graphpad Prism (version 5.00) was used to perform log-rank tests.

Because a large number of genes were tested to determine association with survival, we corrected for multiple testing by estimating the false positive rate. This was done by an in-silico analysis in which a set of 100 genes were randomly mutated across 542 samples (at a population frequency between 2.5-10 %) and we then calculated how many of those were associated with survival using the Cox proportional hazards method. These false positive estimations were made using three different population mutation frequencies (2.5 %, 5 % and 10 %) and was done 50 times for each population mutation frequency. In such analysis, we identified between 1–12 genes that were significantly associated with outcome. For all calculations, *P* < 0.05 was considered statistically significant.

## Results

### Prognostic classification of diffuse gliomas

We analyzed the combined GBM and LGG (low grade glioma) datasets from the TCGA (*n* = 542 samples) and identified 128 genes that are mutated (non-silent mutations only) in ten or more samples, consistent with a population frequency >1.7 % (i.e. 10/542 = 1.8 %). Of these, 57 genes were significantly associated with survival and the list included the well-known favourable prognostic markers *IDH1/2*, 1p19q codeletion, *CIC, FUBP1* and *NOTCH1*. Poor prognostic markers included genetic changes in the *EGFR* locus, *PTEN-*mutations and alt 7/10 (Additional file 1 Table S1). *IDH1 or IDH2-*mutations (collectively referred to in our analysis as *IDH*-mutations unless specifically stated) were associated with the lowest HR (0.10 95 % confidence interval (CI): 0.07-0.14, *P* < 0.0001). Because our aim was to generate a prognostic classification scheme for diffuse gliomas based on molecular aberrations, the gene with lowest HR (i.e. *IDH*-mutations) provided our first molecular prognostic separator for diffuse gliomas.

### Genes associated with prognosis in IDH-wt gliomas

We then screened for prognostic markers separately within *IDH*- wildtype (wt) and *IDH*-mutated gliomas. Within the subset of *IDH*-wt gliomas, we identified 4 genes that, when mutated, were significantly associated with prognosis (Additional file 1: Table S2). However, a relatively large number of tests were performed to identify these genes. To correct for multiple testing, we performed similar analysis on a set of 100 genes that were randomly mutated across the TCGA dataset at a population mutation frequency of 2.5 %, 5 % and 10 %. In such analysis, we identified between 1–12 genes that were significantly associated with outcome. Identification of 4/128 genes associated with survival in *IDH* wt gliomas is therefore within the range of the false positive frequency (1-12 %). By analogy, after Bonferroni correction only one gene (*SLC6A3*) remained significant.

As independent validation is warranted, we screened two additional datasets to confirm the prognostic value of these four genes in *IDH*-wt tumors [6, 17]. Clincal and mutation data are listed in Additional file 1: Tables S3 and S4. In a dataset of anaplastic astrocytomas, mutations in two of these four genes (*PKHD1* and *MUC16*) were identified and in a set of GBMs, mutations in three genes (*MUC16*, *F5* and *PKHD1*) were identified. Unfortunately, the mutation frequency of individual genes was too low to allow for a statistical comparison, and a combined analysis of mutated genes does not show a difference between wt and mutated samples within one dataset. However, when combining survival of both datasets, mutations in any of these genes is associated with poor prognosis (median survival of 0.88 *v.* 1.33 years for mutated and wt samples respectively, *P* = 0.018 HR 3.81, 95 % CI 1.26-11.5). However, because numbers are small, caution should be taken when interpreting these data as it remains possible that the four prognostic genes identified in *IDH*-wt tumors were false positive candidates and do not represent true prognostic genes.

*IDH*-wt diffuse gliomas are often further subdivided into those with trisomy on chromosome 7 combined with LOH of chromosome 10 (alt 7/10) and those without (7/10 wt). It should be noted that, in the TCGA dataset, alt 7/10 does not confer any prognostic information in *IDH*-wt diffuse gliomas (Additional file 1 Table S2). On the gene expression levels alt 7/10 GBMs correlate with “classical” GBMs (or those assigned to IGS-18); 7/10 wt tumors associate with other molecular subtypes (mesenchymal/neural/proneural or IGS-22/IGS-23) [27, 28]. We have therefore screened for prognostic molecular features within the *IDH*-wt, alt 7/10 (‘*molecular classical*’, *n* = 214) and within the *IDH*-wt, 7/10 wt (‘*molecular mesenchymal*’, *n* = 86) diffuse gliomas. Within *molecular classical* gliomas, 10 genes were significantly correlated with survival (Additional file 1: Table S5) and 11 genes within the molecular mesenchymal gliomas (Additional file 1: Table S6). It is interesting to note that *TP53* mutations are associated with a more favourable prognosis in the *molecular classical* gliomas and *PIK3CA* (or combined *PIK3CA* and *PIK3R1*) mutations with poor prognosis in the *molecular mesenchymal* gliomas. Unfortunately, we were unable to validate these results due to an absence of copy number data in the two validation datasets.

It should be noted that pilocytic astrocytomas (PAs, brain tumors with favourable prognosis) may be present among the *IDH*-wt tumors. However, detailed analysis shows that only one of the samples included in this study harboured a genetic profile consistent with PA (TCGA-HT-7691; a diploid genome apart from a tandem duplication on chromosome 7q34 involving the *BRAF* locus), and the survival data for this patient is 0.1 months (patient still alive). Omitting this patient from the analysis will therefore not impact the survival data as presented.

### PI3 kinase pathway mutations are associated with poor survival in molecular astrocytomas

Within *IDH*-mutated diffuse gliomas, we identified 12/128 genes associated with poor survival (Additional file 1: Table S7). Mutations in three and two genes of these were also identified in validation datasets of anaplastic astrocytomas and GBMs respectively [6, 17]. In both datasets, there were too few samples to allow comparison. The absence of a true validation set indicates that caution should be taken as it is possible that the twelve prognostic genes identified in *IDH*-mutant tumors were false positive candidates and do not represent true prognostic genes.

*IDH*-mutated diffuse gliomas are often further subdivided into *molecular astrocytomas* (i.e. those with mutations in *ATRX *and/or *TP53*) and *molecular oligodendrogliomas* (i.e. those with 1p19q codeletion) [16, 23]. It should be noted that these genetic changes by themselves did not reach statistical significance in *IDH*-mutated tumors of the TCGA. This is likely due to the large number of patients alive at time of analysis (205 patients alive out of the 243 *IDH-*mutant glioma patients). We therefore separated IDH-mutated samples into those with *TP53* or *ATRX* mutations (*n* = 151) and those with 1p19q codeletion (*n* = 74). Seventeen samples had neither genetic change and five samples had both.

Within *molecular oligodendrogliomas* we identified 1 out of 128 genes associated with survival (Additional file 1: Table S8). Unfortunately, there are no external datasets to validate this finding.

**Fig. 1:**
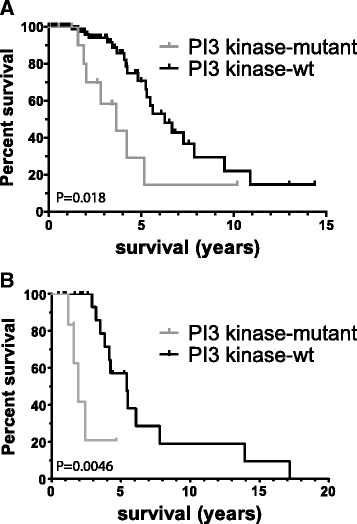
PI3-kinase mutations are prognostic in molecular astrocytomas (diffuse gliomas with *ATRX* and/or *TP53* mutations). **a** Data from TCGA samples (test cohort). Histology and grade of samples presented are listed in Additional file 1: Table S11; **b** Data from two validation cohorts (combined) from astrocytomas [17] and glioblastomas [6]. In both figures, only samples with an *IDH* mutation and *TP53* or *ATRX* were selected. In these molecular astrocytomas, PI3 kinase mutations are prognostic for overall survival. P values indicated are calculated using the Log-rank test. Number of samples analysed are N = 144 (*PIK3* wt) and N = 13 (*PIK3* mutant) for the TCGA cohort and N = 22 (*PIK3* wt) and N = 12 (*PIK3* mutant) for the validation cohort

Within *molecular astrocytomas*, we identified 8 genes associated with survival (Additional file 1: Table S9). *PIK3CA* was one of the genes identified. Interestingly, a similar trend was observed in a highly related gene, *PIK3R1,* HR 2.45 *P* = 0.075 95 % CI 0.91 – 6.56. As *PIK3CA* and *PIK3R1* are highly related (and mutually exclusive) genes within the same PI3-kinase pathway, we combined mutation data into an additional single *PI3-*kinase mutations variable. The median survival in molecular astrocytomas with *PI3-*kinase mutations was 3.7 years *v.* 6.3 years for *PI3*-kinase wt molecular astrocytomas (*P* = 0.02, HR 2.93, 95 % CI 1.16 – 7.38, Fig. 1a). Individual PI3-kinase mutations are listed in Additional file 1: Table S10.* PIK3CA* mutations are missense mutations or in-frame deletions and often affect the known hotspots of the protein (E542, E545 or the C-terminal domain, see [29]). *PIK3R1* mutations are more heterogeneous (in-frame deletions, nonsense, frame-shifts, splice site or missence) not confined to specific hotspots.

To validate the prognostic value of identified genes, we screened an anaplastic astrocytomas dataset and determined survival within defined molecular subtypes of diffuse glioma [17]. Within the *IDH*-mutated and *TP53* or *ATRX* mutated tumors, mutations in four genes out of the 15 identified in the TCGA dataset (*PIK3R1, PKHD1, NEB1,* and *NOTCH2*) were identified. Of these, tumors with *PIK3R1* mutations (*n* = 4) had poorer prognosis than *PIK3R1* wt tumors (*n* = 20), median survival 2.4 and 5.4 years respectively (Additional file 2: Figure S1a). We next downloaded mutation data of a cohort of GBMs [6]. Also in this dataset, we observed a similar poor prognostic trend for PIK3R1 mutations in *IDH*-mutated and *TP53* or *ATRX* mutated GBMs: Tumors with *PIK3R1* mutations (*n* = 2) had poorer prognosis than *PIK3R1* wt tumors (*n* = 2), median survival 1.4 and 5.5 years respectively (Additional file 2: Figure S1b). Although significance was not reached in either of these datasets (perhaps due to the small sample size), a pure molecular classification allows combining both datasets. When this is performed, a median survival of 1.9 *v.* 5.4 years was observed for *PIK3R1* mut and *PIK3R1* wt tumors respectively, HR 17.0, 95 % CI (2.40-121), *P* = 0.0046 (Fig. 1). The fact that *PI3*-kinase mutations showed similar trends in prognosis in three independent datasets, strongly suggests they are prognostic markers for molecular astrocytomas.

### Tumor grade remains prognostic in molecular diffuse glioma subtypes and is associated with mutational load of the tumor

Apart from the pure molecular analysis described above, several clinical and histological parameters are also associated with survival. For example, tumor grade is inversely correlated with patient survival within the defined histological subtypes of diffuse glioma [3]; a correlation that was also present in the TCGA dataset. (Table 1, Additional file 2: Figure S2).

**Table 1:** tumor grade is inversely correlated with patient survival within histological subtypes of diffuse glioma

	Grade II survival (y)	Grade III survival (y)	HR	95 % CI	*P*
Astrocytoma	5.2	3.7	0.27	0.06–1.16	0.078
Oligodendroglioma	7.9	5.2	0.49	0.2–1.2	0.12
Oligoastrocytoma	5.3	6.3	0.26	0.08–0.84	0.024

Survival: median overall survival in years. HR calculated using Cox univariate analysis. HR was calculated grade II vs grade III

**Fig. 2:**
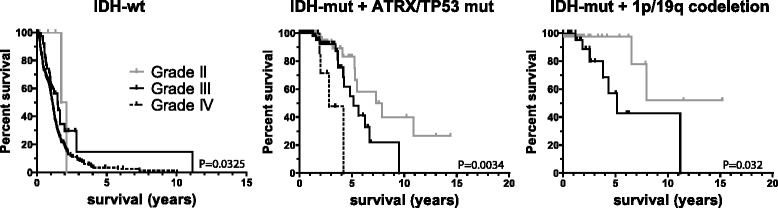
Survival in prognostic molecular subtypes of diffuse glioma stratified by tumor grade. Different subtypes are indicated above each graph. As can be seen, within defined molecular subtypes, tumor grade remains a prognostic factor. Number of samples (grade II, III and IV) for each graph: 10, 42 and 248 (*IDH*-wt); 73, 65 and 13 (*IDH*-mut, *ATRX/TP53* mut); 42, 32 and 0 (*IDH*-mut, 1p19q codeleted). 17 samples were *IDH*-mut but had neither *ATRX/TP53* mutations nor 1p19q codeletion. P values indicated are calculated using the Log-rank test; other test for significance show for IDH-wt tumors a *P* = 0.0092 and for* IDH*-mut, *ATRX/TP53* mut tumors a *P* = 0.0034 (both logrank test for trend); for *IDH*-mut, 1p19q codeleted) a *P* = 0.28 (Gehan Breslow Wilcoxon test). Log rank P values for grade II v. III are 0.43 and 0.063 for *IDH*-wt and *IDH*-mut, *ATRX/TP53* mut tumors respectively

As detailed above, an alternative method for histological classification is to classify gliomas based on their genetic aberrations. Within defined molecular subtypes (i.e. all tumors that harbour mutations in one of the lineage specific genes *IDH, CIC, FUBP1, ATRX, TP53, PTEN, EGFR,* 1p19q codeletion or alt 7/10, frequency listed in Table 2) tumor grade often remained inversely correlated with survival (Additional file 2: Figure S3, Table 3). For example, there were 151 *IDH + ATRX/TP53*-mutated gliomas in the TCGA diffuse glioma datasets of which 73 were of grade II, 65 of grade III and 13 of grade IV (GBM) and median survival was 7.3, 5.2 and 2.8 years (*P* = 0.0024). Similar trends were observed for most other single molecular changes (i.e. selecting samples only on one genetic change, regardless of other molecular changes present). Importantly, tumor grade was a prognostic factor for each of the molecular subtypes identified above: i) *IDH*-wt gliomas; ii) *IDH and TP53 and/or ATRX-*mutated gliomas and; iii) *IDH* and 1p19q codeleted gliomas (Fig. 2).

**Table 2:** Frequency of genetic changes listed per histological subtype and grade

	Low grade	Molecular Oligodendroglioma		Molecular Astrocytoma		Molecular glioblastoma				
	IDH1/IDH2	CIC/FUBP1	LOH 1p19q	ATRX	TP53	EGFR alterations	PTEN	alt 7/10	NF1	N
OD II	95	48	51	28	28	0	2	3	2	65
OD III	82	49	60	18	27	7	2	7	7	45
A II	83	0	0	67	73	0	0	0	0	30
A III	62	1	1	41	65	26	13	26	15	68
OA II	95	14	21	69	74	0	0	2	5	42
OA III	74	10	13	48	58	16	6	16	3	31
GBM	5	0	0	5	30	55	31	71	10	261
N	228	64	74	131	222	170	93	214	44	542

The numbers in the table are percentages of the number of samples mutated (i.e. population frequencies) except the columns listed as N where absolute numbers are given.

**Table 3:** Tumor grade is inversely correlated with survival within molecular subtypes of diffuse glioma

	Grade II		Grade III		Grade IV			
Genes	OS (y)	n	OS (y)	n	OS (y)	n	*P*	P II vs III
*IDH* + *CIC/FUBP1*/LOH 1p19q	"not reached"	47	5.2	34		1	0.040	0.04
*IDH* + *ATRX/TP53*	7.3	73	5.2	70	2.8	13	0.0029	0.069
*EGFR/PTEN*/ alt 7/10	1.9	3	1.5	32	1.2	211	0.13	0.5
*NF*1	2.1	3	1.9	14	1	27	0.034	NA

IDH + CIC/FUBP1/LOH 1p19q refers to mutations in IDH plus any of the subsequent genes, similar for *IDH* + *ATRX/TP5* *3*. Statistical tests were performed using a Chi-square test. OS refers to median overall survival in years. Frequency comparisons were done between grade II, III and IV. Exceptions were made for genes with too few/no data in one of the grades (e.g. there are no grade IV tumors with 1p19q codeletion). Therefore, the P value for NF1 is based on comparison between grade III and IV and the P value for *IDH* + *CIC/FUBP1*/LOH 1p19q is based on a comparison between grade II and III

Because tumor grade was associated with patient survival, we further analysed the TCGA dataset to identify the molecular correlates of tumor grade. When screening for mutations that occur at different frequencies between grade II and III diffuse gliomas, the only genes identified were the lineage specific genetic changes (*IDH*, *CIC*, 1p19q co-deletion, *ATRX*, *EGFR*, and alt 7/10). These genes are listed in Table 2 and such a higher rate (where the frequency of mutations in grade II > grade III) has been observed in other studies [30, 31] (although other studies did not find such a difference [14]). Perhaps the most striking difference between tumors of different grade however was the total number of genetic changes (the mutational load). For example, the average number of non-silent (i.e. those that result in a change in the primary protein sequence) genetic changes in grade II astrocytomas was 18.8 ± 13.1 (*n* = 30), in grade III astrocytomas it was 36.8 ± 47.6 (*n* = 68), *P* = 0.0050 (Table 4). This increase in ‘mutational load’ was also observed within molecular subtypes of diffuse glioma and is listed in Table 5. For example, the mutational load of *ATRX* mutated gliomas increased from 21.6 ± 10.3 and 26.0 ± 11.2 to 65.4 ± 40.1 mutations per sample (*P* < 0.0001) for grade II, III and IV gliomas respectively.

**Table 4:** Tumor grade is correlated with mutational load within histological subtypes of diffuse glioma

	Grade II	Grade III	Grade IV	*P*
Oligodendroglioma	21.8 ± 10.3 (65)	28.1 ± 13.5 (45)		0.011
Astrocytoma	18.8 ± 13.1 (30)	36.8 ± 47.6 (68)		0.0050
Oligoastrocytoma	20 ± 9 (42)	29.3 ± 14.3 (31)		0.0025
GBM			57.3 ± 19.9 (261)	

Values are listed as the average number of non-silent mutations +/− SD (number of tumors analyzed. P values were calculated using an anova

**Table 5:** Tumor grade is correlated with mutational load within molecular subtypes of diffuse glioma

	Grade II	Grade III	Grade IV	*P*	P II v. III
Overall	20.6 ± 10.6 (137)	32.4 ± 34.3 (144)	57.3 ± 19.9 (261)	<0.0001	
*IDH1/IDH2*	21.1 ± 10.1 (127)	26.7 ± 12.1 (102)	52 ± 22.1 (13)	<0.0001	0.00023
*CIC/FUBP1*	21.9 ± 10.3 (37)	28 ± 10.7 (26)		0.030	
LOH 1p19q	21.7 ± 10.1 (42)	28.2 ± 10.2 (32)		0.0081	
*ATRX*	21.6 ± 10.3 (67)	26 ± 11.2 (51)	65.4 ± 40.1 (14)	<0.0001	0.034
*TP53*	21.4 ± 10.2 (71)	33 ± 46.1 (74)	60.5 ± 23 (78)	<0.0001	0.038
*EGFR*		41.9 ± 12.7 (15)	60.3 ± 16.6 (69)	<0.0001	
*PTEN*		42.8 ± 10.8 (12)	62.7 ± 21.3 (80)	<0.0001	
alt 7/10	24 ± 10.6 (3)	43.5 ± 10.1 (26)	59.6 ± 16.7 (185)	<0.0001	
*NF1*	12 ± 7.2 (3)	57.6 ± 101.6 (14)	56.6 ± 15.5 (27)	0.97	

Values are listed as the average number of non-silent mutations +/− SD (number of tumors analyzed). Alt 7/10: Trisomy chromosome 7 and LOH of chromosome 10. P values were calculated using an anova. P II *v.* III indicates significance of grade II *v.* grade III tumors based on a *T*-test. Total number of cases analysed = 542

### The mutational load is associated with patient age

**Fig. 3:**
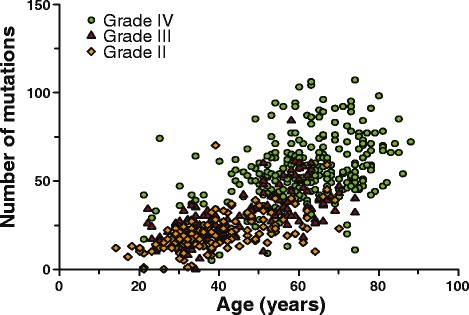
Correlation between patient age and mutational load in diffuse gliomas. The number of non-silent genetic changes increases with patient age. This increase is irrespective of histological subtype (not shown) or tumor grade. Two samples (out of the 542 analyzed) with a high mutational load fall outside the y-axis limit in this figure: a grade III astrocytoma, age 47, mutational load 408 and a GBM, age 45, mutational load 181

Because age is a well-known prognostic factor in diffuse glioma patients, we included age in the analysis. Similar to previously reported [1, 32, 33], grade II tumors occur in patients that were younger than those with grade III or grade IV tumors, 39.6 ± 12.5 (*n* = 137), 45.6 ± 13.5 (*n* = 144) and 61.3 ± 13.0 (*n* = 261) years respectively (average ± standard deviation (SD), *P* < 0.0001 for any comparison, ANOVA). As patient age and tumor grade were correlated, and tumor grade was correlated to the mutational load, it is not surprizing that age was also correlated with the mutational load of the tumor (Fig. 3). This correlation was observed not only in the entire dataset but also within histologically and molecularly defined subtypes (Tables 5 and 6). Indeed, when analysing the type of mutations that occur in the TCGA dataset, a large proportion (2962/9281, 32 %) of all mutations were C > T transitions in the sequence xCG (where x represents any nucleotide). Only 4/96 possible combinations would lead to this specific mutation, and this type of signature has been identified as an age related mutation signature [34].

**Table 6:** Tumor grade is correlated with patient age within molecular subtypes of diffuse glioma

	Grade II	Grade III	Grade IV	*P*	P II v. III
Overall	39.6 ± 12.5 (137)	45.6 ± 13.5 (144)	61.3 ± 13 (261)	<0.0001	
*IDH1/IDH2*	39.6 ± 12.3 (127)	42 ± 12.1 (102)	39.6 ± 15.7 (13)	0.32	0.14
*CIC/FUBP1*	42.3 ± 13.4 (37)	48 ± 10.8 (26)		0.065	0.065
LOH 1p19q	42 ± 12.4 (42)	49.4 ± 11.8 (32)		0.012	0.012
*ATRX*	37.4 ± 11.9 (67)	38.1 ± 11.3 (51)	41.6 ± 17.2 (14)	0.30	0.74
*TP53*	37.2 ± 11.8 (71)	39.9 ± 11.7 (74)	59.2 ± 15.5 (78)	<0.0001	0.18
*EGFR*		61.7 ± 7.5 (15)	61.2 ± 11.7 (69)	0.84	
*PTEN*		56.8 ± 10.5 (12)	62.8 ± 11.9 (80)	0.092	
alt 7/10	49.7 ± 8.3 (3)	59.4 ± 6.8 (26)	62.8 ± 10.8 (185)	0.015	
*NF1*	51 ± 18.4 (3)	43.7 ± 12.7 (14)	64.4 ± 13.2 (27)	0.00050	

Values are listed as the mean patient age +/− standard deviation (number of tumors analysed). P values were calculated using an anova. P II *v.* III indicates significance of grade II *v.* grade III tumors based on a *T*-test. Total number of cases analysed = 542

Univariate analysis confirmed that histology (oligoastrocytoma vs. oligodendroglioma: P = 0.41 HR 1.33 95 % CI 0.68-2.61; astrocytoma vs. oligodendroglioma: *P* = 0.0029 HR 2.52 95 % CI 1.37-4.63; GBM vs oligodendroglioma: *P* = <0.0001 HR 10.6 95 % CI 6.47-17.3), tumor grade (grade III vs. II: *P* = 0.0001, HR 3.14 95%CI 1.76-5.60; grade IV vs. II: *P* < 0.0001, HR 14.4 95%CI 8.48-24.5), the number of mutations (*P* < 0.00001, HR 4.52, 95%CI 3.42 – 5.97) and patient age (*P* < 0.00001, HR 5.51, 95%CI 4.03 – 7.54) were associated with patient overall survival. In a multivariate analysis, the number of mutations remained a significant prognostic factor when including histology and tumor grade in the analysis. However, when the multivariate analysis also included patient age, the number of mutations was no longer a significant prognostic marker (Table 7). Similar results were obtained when performing multivariate analysis within defined molecular subtypes (mutations in *IDH*, *CIC* or *FUBP1*, *TP53*, *EGFR*, *PTEN*, *NF1* or trisomy of Chr7 combined with LOH of Chr 10 or 1p19q codeletion), data not shown. Therefore, patient age appears to be stronger associated with patient survival than mutational load.

**Table 7:** multivariate Cox analysis of prognostic markers for overall survival in diffuse glioma patients

	HR	*P* value	95 % CI
Histology	1.00		
Oligoastrocytoma vs. oligodendroglioma	1.53	0.22	0.78–3.02
Astrocytoma vs. oligodendroglioma	2.19	0.015	1.17–4.11
Grade	1.00		
III vs. II	2.46	0.0040	1.33–4.54
IV vs. II^*^	6.41	<0.0001	2.11–4.57
Age	1.00		
> 50 vs. ≤50	3.10	<0.0001	2.11–4.57
Mutational load	1.00		
> 40 vs. ≤ 40	0.69	0.066	0.47–1.03

A total of 542 samples were analyzed for this table. HR: Hazard Rate; CI: Confidence interval. Grade levels were 2, 3 and 4. Three histology levels were used (oligodendroglioma, oligoastrocytoma and astrocytoma), GBMs were categorized as astrocytomas

## Discussion and conclusions

**Fig. 4:**
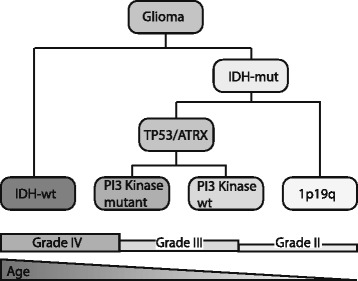
Proposed scheme for the prognostic classification of diffuse gliomas. Diffuse gliomas are first stratified based on their *IDH*-mutation status. Further classification is based on the *ATRX* and/or *TP53* mutation status or determining 1p19q codeletion (these changes are mutually exclusive). Within the *ATRX* and/or *TP53* mutated samples, mutations in *PI3* kinase genes *PIK3CA* and *PIK3R1* are associated with poor prognosis. It should be noted that there are genetic changes that associate with each molecular subtype (like *EGFR* amplification with *IDH*-wt tumors). They are however, not important for prognostic classification and may occur in several molecular subtypes. For example, PI3K mutations occur in all molecular subtypes but are only significantly prognostic in *IDH*-mutated, *TP53/ATRX* mutated diffuse gliomas). Additional prognostic factors include tumor grade and patient age, both of which are correlated to the mutational load of the tumor and are listed below the classification scheme. These additional markers are often correlated to the mutational profile of the tumors: Patients with *IDH*-wt tumors are often older and most are diagnosed as grade IV. ATRX/TP53 indicates mutation of either/both genes; 1p19q indicates codeletion of these chromosomal arms

In this study, we have aimed to identify genetic changes associated with patient prognosis within defined histological and molecular subtypes of diffuse glioma by analysing the TCGA glioma datasets. Our analysis shows that diffuse gliomas are first classified based on their *IDH*-mutation status. Further stratification into molecular oligodendrogliomas and molecular astrocytomas involves determining the *ATRX* and/or *TP53* mutation status or determining 1p19q codeletion (these changes are mutually exclusive). Within molecular astrocytomas, mutations in *PI3* kinase genes *PIK3CA* and *PIK3R1* are likely to be associated with poor prognosis. Additional prognostic factors include tumor grade and patient age, both of which are correlated to the mutational load of the tumor. A scheme for the prognostic classification is proposed in Fig. 4.

A novel prognostic marker identified by current analysis are *PI3* kinase mutations. Such mutations are frequently observed in various cancer types including diffuse gliomas [29, 35]. They act as lipid kinase downstream of various receptor tyrosine kinases, ultimately resulting in activation of signalling cascades involved in cell growth and proliferation, survival and migration [36]. It has been speculated that, as *PI3* kinase mutations are frequently observed in diffuse gliomas, specific inhibitors may provide clinical benefit for *PI3* kinase mutated diffuse glioma patients [37]. Here we show that *PI3* kinase mutations also act as prognostic markers for molecular astrocytoma patients, providing the first evidence to demonstrate they are associated with poor outcome within a defined glioma subtype.

Our analysis also shows that grade is associated with mutational load of the tumor. This is an interesting observation as the mutational load may provide a biological explanation for tumor grade. Even if only a subset of the affected genes contributes to gliomagenesis and/or progression, an increase in mutational load would increase tumor aggressiveness. Indeed, several studies on genes mutated at a low population frequency (‘low frequency genes’) have demonstrated that they can contribute to tumor formation or progression [38–43]. In a larger study, we have shown that many (but not all) mutations in low frequency genes affect their functional property [44]. In addition, mouse experiments have demonstrated that the age of the cells in which a glioma is generated largely determines their survival and not the age of the mouse into which the tumor is transplanted. These data argue for an intrinsic (age-related) property of the tumor initiating cell, perhaps mutational load [45]. Interestingly however, in a multivariate analysis, the mutational load is no longer a significant prognostic marker when patient age is included. The mutational load therefore cannot fully explain the increased aggressiveness of tumors of higher grade.

Our analysis also indicates that each malignancy grade is associated with a different prognosis within molecularly similar tumors. These results appear to be in contrast with a recent publication that failed to identify differences in survival between grade II and III *IDH*-mutant astrocytic tumors [24]. Similarly, a second paper found only a modest impact of tumor grade in *IDH*-mutated grade II and III gliomas [25]. However, our analysis included all tumor grades (II-IV) whereas those studies focussed only on grade II and III. In addition, our analysis did not preselect for a specific histological subtype.

It is often reported that *IDH1* mutated GBMs have a better prognosis than *IDH1*-wt gliomas [6, 12]. The analysis presented here (using TCGA data) also shows that *IDH1* mutated grade IV tumors have a poorer prognosis than *IDH1*-mutated lower grade gliomas, which has also been observed in other studies. For example, *IDH1* mutated GBMs have a survival in the range of 24–30 months whereas IDH1 mutated grade III astrocytic tumours, median survival is significantly longer surpassing 50–60 months [7, 12] and similarly, *IDH1*-wt GBMs have median survival of 11–15 months whereas *IDH1*-wt grade III astrocytic tumours have a median survival in the range of 21 months [12]. Here we show that the correlation between grade and prognosis is also true for other molecularly similar tumors. These data therefore argue for inclusion of tumor grade as prognostic factor when molecularly classifying diffuse gliomas and indicate that molecularly similar tumors of different grade should not be treated identical.

## Additional files

Additional file 1:
**Table S1.** Genetic changes associated with survival in the entire TCGA (GBM and LGG) datasets. **Table S2.** Genetic changes associated with survival in IDH-wt gliomas. **Table S3.** Validation data from Parsons et al. **Table S4.** Validation data from Killela et al. **Table S5.** Genetic changes associated with survival in IDH-wt, alt 7/10. **Table S6.** Genetic changes associated with survival in IDH-wt, 7/10 wt. **Table S7.** Genetic changes associated with survival in IDH-mutant gliomas. **Table S8.** Genetic changes associated with survival in IDH and 1p19q. **Table S9.** Genetic changes associated with survival in IDH and TP53/ATRX mutated gliomas. **Table S10. Table S11.** Histological subtypes in molecular astrocytomas stratified by PI3-kinase mutations. (XLSX 145 kb) Additional file 2:
**Figure S1.** *PI3-*kinase mutations are prognostic for survival in independent datasets. **Figure S2.** Survival in histological subtypes of glioma stratified by tumor grade of samples included in the TCGA dataset. **Figure S3.** Overall survival in distinct molecular subtypes of glioma stratified by tumor grade of samples included in the TCGA dataset. (DOCX 452 kb)
