# Supplementary material for: PI3 kinase mutations and mutational load as poor prognostic markers in diffuse glioma patients
Source: Acta Neuropathol Commun. 2015 Dec 23;3:88. doi: 10.1186/s40478-015-0265-4 (PMC4690424; doi:10.1186/s40478-015-0265-4)
Supplement: Additional file 2: — Figure S1. PI3-kinase mutations are prognostic for survival in independent datasets. Figure S2. Survival in histological subtypes of glioma stratified by tumor grade of samples included in the TCGA dataset. Figure S3. Overall survival in distinct molecular subtypes of glioma stratified by tumor grade of samples included in the TCGA dataset. (DOCX 452 kb) [file 40478_2015_265_MOESM2_ESM.docx]

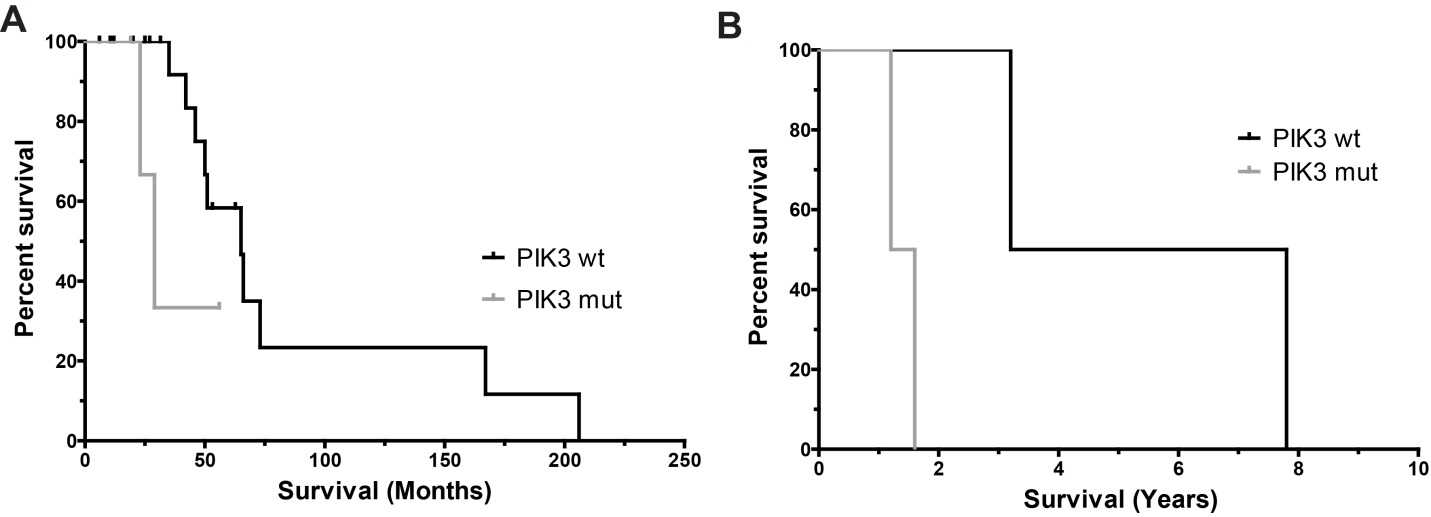


P=0.13

P=0.090

Figure S1: *PI3-*kinase mutations are prognostic for survival in independent datasets. Validation showing that *PI3-*kinase mutations are prognostic for survival in a dataset of astrocytomas (A) and glioblastomas (B, n=4) after selecting for tumors for *IDH1* and *TP53/ATRX* mutations ([1](#_ENREF_1), [2](#_ENREF_2)). *PI3-*kinase mutations were found in 4/24 and 2/4 samples in the astrocytoma and glioblastoma datasets.


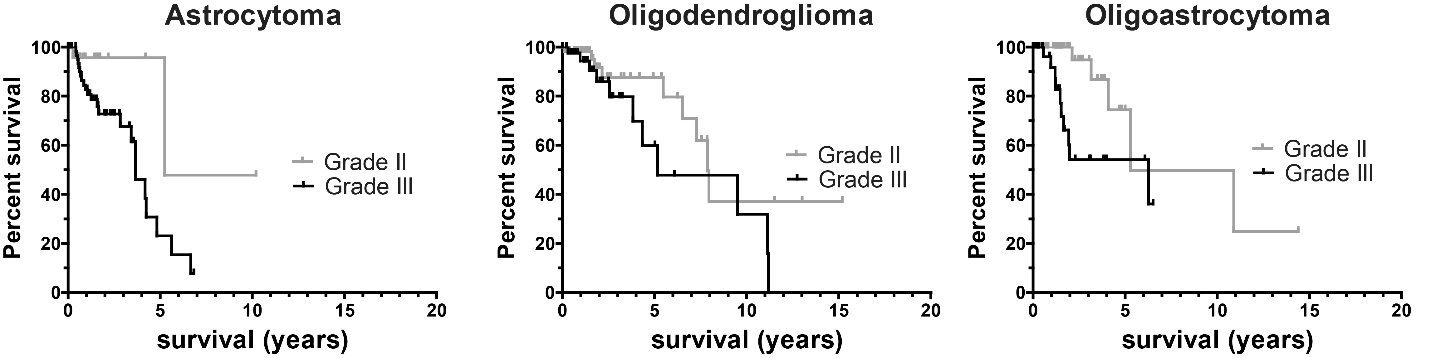


P=0.016

P=0.11

P=0.053

Figure S2: Survival in histological subtypes of glioma stratified by tumor grade of samples included in the TCGA dataset. Median overall survival (OS) for astrocytomas was 5.2 and 3.7 years for grade II (n=30) and grade III (n=68). Median OS for oligodendrogliomas was 7.9 and 5.2 years for grade II (n=65) and grade III (n=45). Median OS for oligoastrocytomas was 5.3 and 6.3 years for grade II (n=42) and grade III (n=31), all P values stated in the figures are calculated using a Log-rank test. A Gehan-Breslow-Wilcoxon test, which gives more weight to early events, was also performed using Graphpad Prism software and yields P values of 0.19, 0.28 and 0.0013 for astrocytomas, oligodendrogliomas and oligoastrocytomas respectively.


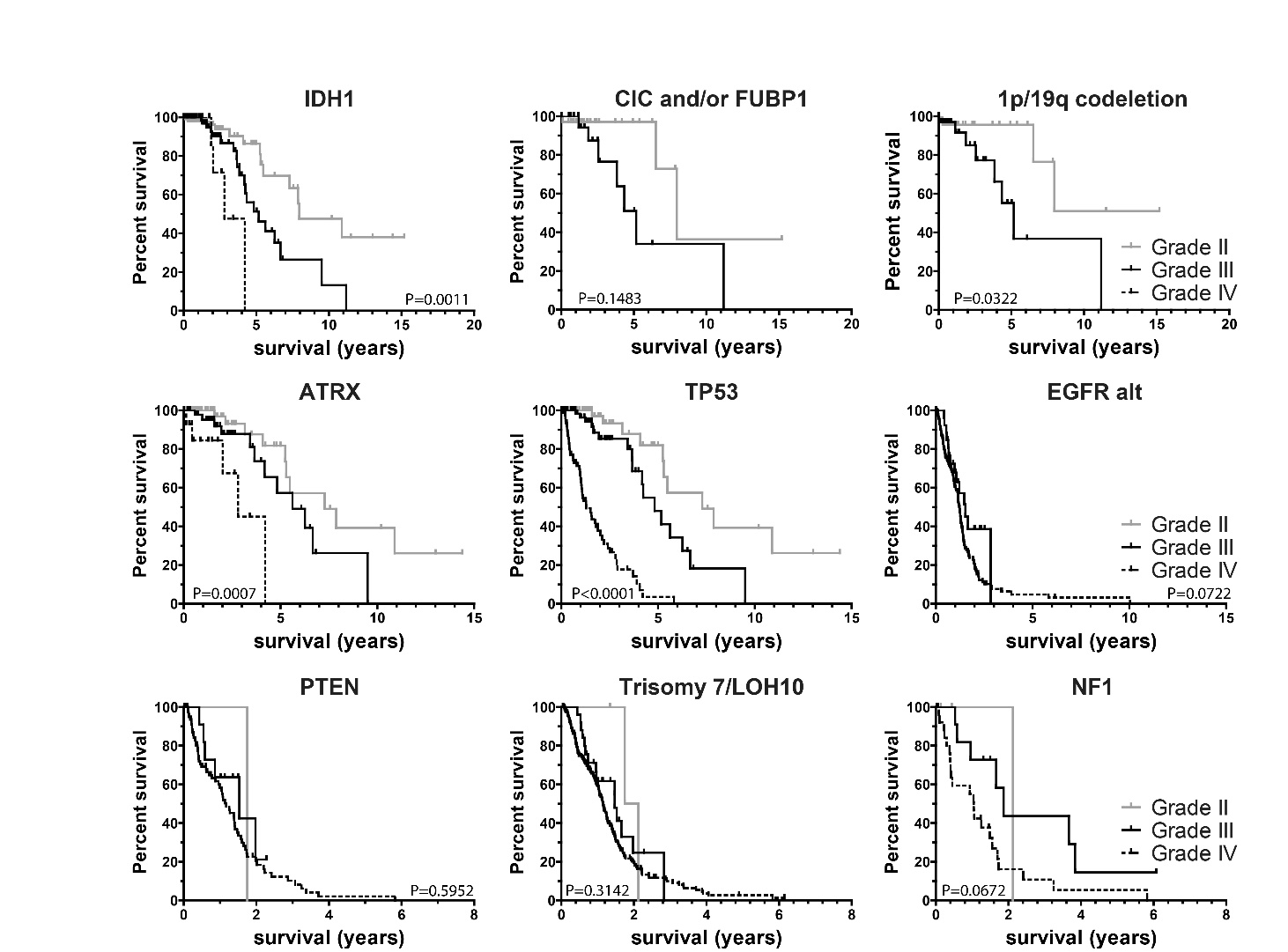


Figure S3: Overall survival in distinct molecular subtypes of glioma stratified by tumor grade of samples included in the TCGA dataset. As can be seen, within most defined molecular subtypes, tumor grade remains a prognostic factor. P values were calculated using a log rank test. Number of samples (grade II, III and IV) for each graph: 119, 96 and 13 (IDH1); 37, 26 and 1 (CIC and/or FUBP1); 42, 32 and 0 (1p/19q codeletion); 67, 50 and 14 (ATRX); 71, 73 and 78 (TP53); 0, 26 and 144 (EGFR); 1, 12 and 80 (PTEN); 3, 26 and 185 (trisomy 7/LOH10); 3, 14 and 27 (NF1). Most cases with CIC/FUBP1, 1p/19q codeletion, ATRX or TP53, also have an IDH mutation

**References**

**1**. Killela PJ, Pirozzi CJ, Reitman ZJ, Jones S, Rasheed BA, Lipp E, et al. The genetic landscape of anaplastic astrocytoma. Oncotarget 2014;5:1452-7.

**2**. Parsons DW, Jones S, Zhang X, Lin JC, Leary RJ, Angenendt P, et al. An integrated genomic analysis of human glioblastoma multiforme. Science 2008;321:1807-12.
